# Narrow-Front Loop Migration in a Population of the Common Cuckoo *Cuculus canorus*, as Revealed by Satellite Telemetry

**DOI:** 10.1371/journal.pone.0083515

**Published:** 2014-01-08

**Authors:** Mikkel Willemoes, Roine Strandberg, Raymond H. G. Klaassen, Anders P. Tøttrup, Yannis Vardanis, Paul W. Howey, Kasper Thorup, Martin Wikelski, Thomas Alerstam

**Affiliations:** 1 Center for Macroecology, Evolution and Climate, Natural History Museum of Denmark, University of Copenhagen, Copenhagen, Denmark; 2 Department of Biology, Lund University, Lund, Sweden; 3 Dutch Montagu's Harrier foundation and Animal Ecology Group, Centre for Ecological and Evolutionary Studies, Groningen University, Groningen, the Netherlands; 4 Microwave Telemetry Inc, Columbia Maryland, United States of America; 5 Max Planck Institute for Ornithology, Department of Migration & Immuno-ecology, Radolfzell am Bodensee, Germany; 6 Department of Biology, University of Konstanz, Konstanz, Germany; Hungarian Academy of Sciences, Hungary

## Abstract

Narrow migration corridors known in diurnal, social migrants such as raptors, storks and geese are thought to be caused by topographical leading line effects in combination with learning detailed routes across generations. Here, we document narrow-front migration in a nocturnal, solitary migrant, the common cuckoo *Cuculus canorus*, using satellite telemetry. We tracked the migration of adult cuckoos from the breeding grounds in southern Scandinavia (n = 8), to wintering sites in south-western Central Africa (n = 6) and back to the breeding grounds (n = 3). Migration patterns were very complex; in addition to the breeding and wintering sites, six different stopover sites were identified during the 16,000 km annual route that formed a large-scale clockwise loop. Despite this complexity, individuals showed surprisingly similar migration patterns, with very little variation between routes. We compared observed tracks with simulated routes based on vector orientation (with and without effects of barriers on orientation and survival). Observed distances between routes were often significantly smaller than expected if the routes were established on the basis of an innate vector orientation programme. Average distance between individuals in eastern Sahel after having migrated more than 5,000 km for example, was merely 164 km. This implies that more sophisticated inherent guiding mechanisms, possibly involving elements of intermediate goal area navigation or more elaborate external cues, are necessary to explain the complex narrow-front migration pattern observed for the cuckoos in this study.

## Introduction

Every year billions of birds migrate between Europe and sub-Saharan Africa [1], [2]. Especially for the smaller, nocturnal migrants, little is known about population specific migration schedules, migration routes, stopover sites and non-breeding ranges [3]. For some diurnally migrating birds, and particularly for soaring migrants utilising thermal updrafts, it is well known that populations follow specific and narrow migration corridors. Typically these birds avoid large water bodies and high mountains, resulting in funnel-shaped flyways converging at specific points [4]. Well known migration hot spots, like the Strait of Gibraltar, Bosporus and the Isthmus of Panama, are good examples of such convergence points, although it is not clear how many birds do not use these specific concentration points. For many nocturnal, solitary migrants, for example most songbirds, migration corridors are generally much less condensed (often several hundred kilometres broad as seen from e.g. bird ringing atlases of Scandinavian birds [5], [6]). In social migrants, young birds on their first migration receive guidance from experienced birds as in day-migrating geese, storks, cranes [7], [8], [9] and possibly raptors [10]. In contrast, young solitary migrants are supposedly only led by their innate migration programme. It is generally believed that the innate migration programme is based on a simple vector orientation principle (a clock-and-compass strategy) [4], [11], perhaps with some external influences. An additional component of true navigation is often considered unlikely among naïve birds on their first migratory journey (but see [12], [13], [14]). As the scatter between individuals is always increasing with distance travelled in migration routes resulting from a clock-and-compass strategy [15], some external factors (topography, weather, habitat etc) or the possibility of goal area navigation are necessary to explain convergence of migration tracks [16], [17].

Currently, full migration patterns of many species of birds are being revealed (e.g. [18], [19], [20]), though large diurnally migrating species are still over-represented in this field [21]. Nocturnal, solitary migrants are typically small and it has so far not been possible to track these migrants using accurate methods such as satellite telemetry or GPS. Here, we present the first migration tracks of the common cuckoo *Cuculus canorus*, which is a primarily nocturnal, solitary migrant [2], [22], [23]. The common cuckoo is an obligate nest parasite and young cuckoos are raised by a host species. Because the adults leave the breeding grounds well before the young, it can be ruled out that young, naïve cuckoos learn from experienced adults [24]. Hence, cuckoo migration is the ultimate example of a genetic basis of migration patterns. However, little is known about its migration. Cuckoos are rarely seen in high concentrations on the typical migration hotspots along the Mediterranean coast and they are thought to migrate over a broad front and possibly overfly northern Africa without stopping [24], [25]. Ringing has only provided sparse information of the migration route. From southern Scandinavia, two birds ringed in Denmark were recovered in Italy in spring [5] and four birds ringed in Sweden were recovered around the Mediterranean in autumn; two in eastern Italy, one in Greece and one in north-western Egypt [26]. No ringing recoveries from Scandinavian cuckoos are available from sub-Saharan Africa.

We describe the entire annual migration cycle of adult common cuckoos from their breeding sites in southern Scandinavia to the winter grounds in south-western Central Africa and back. The tracks revealed that birds from this population were migrating in a large clockwise loop during their annual cycle, with the different individuals following closely similar routes using several stopover areas in both temperate and tropical zones without diverging from each other to any large degree. This similarity between individual routes in such a complex geographic migration pattern seems to be difficult to reconcile with the assumption that individual migration patterns have been established as a result of an innate vector orientation programme during the first annual migratory journey.

For adult birds, it is assumed that they have learnt about suitable stopover and wintering areas during their first journey as young birds, allowing them to navigate back to these familiar areas during subsequent journeys of their lives [4], [27]. This means that the spatial variation between adult individuals to a large degree is expected to reflect the scatter caused by the vector orientation during the individuals' first journeys, while consistency within individuals in their successive journeys, showing movements towards the same stopover/wintering/breeding areas, reflects the learned ability of navigation/homing.

According to the principles of vector orientation alone, we should always expect the scatter between the routes of different individuals to increase with distance along the migration route. However, there are three main factors that may limit or reduce this increase in spatial scatter between individuals. (*i*) The scatter between individuals will increase with distance at a slower rate with increasing number of orientation steps/vectors (or with shorter step/vector lengths) used by migrants to cover a certain distance (with the successive steps/vectors drawn from a circular distribution with a given mean and variation). Hence, the frontal geographic scatter is expected to be approximately proportional to (n)^−½^, where n is the number of orientation steps to reach a certain total distance (cf. [13], [14], [15], [28], [29]). (*ii*) If vector orientation is combined with a response to topographical features (large water bodies, mountains or other habitats acting as ecological barriers) this will have the effect of adjusting individual routes towards more favourable habitats and away from large barriers, which will decrease the scatter between routes. (*iii*) Differential survival of juveniles on their first journey could have the effect that only individuals following specific favourable routes by their vector orientation programme will survive. This may lead to a reduced spatial scatter among adult individuals as a result of mortality of juveniles travelling outside the most favourable migration corridor. Of course, the mortality loss of juveniles must be substantial to explain a very narrow corridor for the surviving population of adult individuals.

In order to investigate if vector orientation, alone or in combination with the effects of topographical response and differential survival, can explain the observed pattern of complex loop migration of the cuckoos, we compare observed tracks with tracks simulated according to a clock-and-compass migration programme based on the migratory behaviour shown by the tracked birds. Particularly we investigate if the concentration of tracks is similar between simulated and observed routes.

We demonstrate that it is highly unlikely that the complex loop migration of southern Scandinavian cuckoos is generated by a vector orientation programme, not even if such a programme is combined with inherent responses to topographical features and with strong mortality of juveniles on the flanks of the primary loop migration corridor. We infer that these migrants are likely to rely on more sophisticated inherent instructions along their migration route, to allow the evolution of complex and narrowly defined population-specific migration routes.

## Methods

### Ethics statement

Animal work in Denmark was approved by the Danish Nature Agency by permission to the Copenhagen Bird Ringing Centre (J.nr. SN 302-009). In Sweden, the study was approved by the Ethical Committee for Animal Experiments in Malmö/Lund (M112-09). The Danish Nature Agency gave permission to work on their land in Denmark.

### Tracking

Eight adult common cuckoos, five males and three females, were tagged on the breeding grounds in eastern Denmark and southern Sweden (55.68–56.05°N, 12.30–14.53°E) and followed from the breeding season in 2010 to the breeding season in 2011 or until transmission stopped. Three birds transmitted the entire annual cycle returning to the breeding grounds. One stopped transmitting in north-central Europe, one in the southern Sahara, one in the winter quarters, one in West Africa and one in northern Africa north of the Sahara.

The birds were caught using mist nets and tape lure in wet land areas with plenty of bushes (primarily *Salix spp*) and heavy reed *Phragmites australis* growth. Cuckoos from these habitats and in this region are typically using Eurasian reed warbler *acrocephalus scirpaceus* as a host species. The tagged birds were sexed using plumage, biometric and voice characters. The transmitters, Solar PTT-100s (Microwave Telemetry Inc.), were fitted to the birds as a back-pack using a body harness made from a 2 mm braided nylon string. The weight of the transmitters was 5 g corresponding on average to 4.3% (3.7–5.0%) of the body weight of the cuckoos. Transmission was scheduled on a 10 hours on 48 hours off duty cycle.

Geographical positions of the transmitters were obtained from ARGOS/CLS Service Argos [30]. Position estimates from ARGOS are assigned to a location quality class (3-0, A–B and Z; 3 has highest and Z lowest accuracy). We excluded all positions of class Z. During transmission periods, we determined whether a bird was travelling or stationary. A bird was considered stationary, if distances moved were small (<10 km; high quality class positions) or in random directions (low quality positions). For stationary birds, only the highest quality position from each 10 h transmission period was included. For travelling birds, all positions during the 10 h transmission periods were used for analyses. For several transmission cycles no positions were obtained, especially in Europe during autumn where the birds passed through an area with well known poor satellite reception, probably due to a high level of background noise in this area [31].

Staging periods were defined as those lasting a minimum of 5 days. To give an overview of the migration pattern, we defined major migration steps and staging areas. Migration steps were defined as steps between staging areas shown by at least half of the individuals (ESM table S1). Individual staging areas were considered the same if they were in the same overall region and were in the same stage of the migration (e.g. staging areas in Hungary and Bulgaria were considered the same staging area since they were all in south-eastern Europe and following a stopover in northern Europe). In some cases, we lacked the positions necessary to determine whether a bird used a specific stopover area. For the same reason, departure and arrival dates at stopover areas are in some cases uncertain.

We defined the mean stopover position as the average of longitude and latitude for all birds using that stopover area. The distance between the birds at each stopover was calculated as the average of the distances from each tracked individual to the mean position in that stopover area.

### Simulations

We simulated several possible migration scenarios based on a clock-and-compass migration system (Table 1). The clock-and-compass system was modelled using vector summation [12], [13], [14], [15] according to each specific scenario based on the directional concentration and step lengths estimated from the set of migration vectors identified above (the steps between stopovers). In other studies using vector summation, each migration step is normally modelled as a vector with a specified length and a direction taken randomly from a directional distribution, typically the von Mises distribution [14], [32], [33]. We used this approach when simulating migration with short steps, and used random recombination of the actual observed vectors when simulating long steps (see below).

**Table 1:** Description of the different migration scenarios simulated. See Methods for more details.

Scenario	Name	Topography effects	Description
(i)	LONG	Not included	Migration simulated as a clock-and-compass strategy with single steps between each stopover and winter sites (8 steps in total). Each step is drawn randomly between the vectors observed in the tracked birds. This scenario is valid both for adults following the same route as they did as juveniles and for adults using a clock-and-compass strategy directly
(ii)	SHORT	Not included	Same as (i) LONG above, except that the step length is 100 km. Directions are drawn randomly from a circular distribution that fits the concentrations at each stopover.
(iii)	SHORT_Mort	Direct mortality	Migration of adult birds with birds crossing mountains or ocean dying, i.e. tracks are discontinued after crossing. Otherwise as (ii) SHORT.
(iv)	SHORT_Dir_Change	Change of migration direction	Migration simulated as a clock-and-compass strategy with birds encountering impassable mountains or ocean choosing another random direction to circumvent it if possible. This scenario is valid both for adults following the same route as they did as juveniles and for adults using a clock-and-compass strategy directly
(v)	LONG_Juv_Mort	Juvenile mortality	Migration of adult birds following the routes followed as juveniles but with juvenile mortality taken into account. Thus, juvenile birds crossing mountains or ocean are assumed to die and thus, these tracks are removed. Step length as in (i) LONG.
(vi)	SHORT_Juv_Mort	Juvenile mortality	As above (v) LONG-Juv-Mort, except that steps are modelled as in (ii) SHORT.

We investigated if the observed narrow loop migration pattern could be explained by a clock-and-compass system, using the following migration scenarios (Table 1): (*i*) Adults using single vector steps between each stopover and winter site, not considering topography. (*ii*) As (*i*), except that the vector step length was 100 km (i.e. many steps between each stopover). (*iii*) Migration of adult birds, but with mortality resulting from barrier crossing taken into account. Since mortality also occurred among the adults we tracked, we did not remove the entire route, but only the part that comes after a barrier. Step length was 100 km. (*iv*) Adults encountering impassable barriers (mountains or ocean) choose another random direction to circumvent it if possible. Step length was 100 km. (*v*) Adult birds following the routes arisen from juvenile vector orientation, but with juvenile mortality resulting from barrier crossing implemented. As the mortality occurs prior to our tracking (during the birds' first migration cycle as juveniles), entire maladaptive routes were removed. This was modelled with single steps between stopovers as in (*i*). (*vi*) As (*v*), except that step length was 100 km.

#### Simulations excluding effects of barriers: models (i) and (ii)

Our basic simulations assumed that migration routes were established by the use of a clock-and-compass strategy between the successive stopover and winter sites. Under this assumption, we fitted two models. In the first, the distance between stopovers was covered in one single step. For this simulation, the vector summation was done by simply using the vectors that the birds used for travelling between stopovers, i.e. random combinations of the individual vectors recorded for each of the eight main steps of the annual migration loop. In the second model, we used a more typical step length of 100 km [12], [14]. Shorter steps result in less scattered routes, but as it is unlikely that daily migration steps (flight steps during a night) were shorter than 100 km (this data) or that orientation is re-established more frequently than at each daily flight step [34], [35], we consider this a conservative approach. The concentration of the directional distribution to be used with the smaller step length was found as the one in which vector addition resulted in a mean vector with concentration and length equal to the mean step vector observed for tracked birds at each of the eight different main steps between stopover areas. For each model, we simulated 1000 tracks.

#### Simulation with removal of part of the track after reaching barriers: model (iii)

The topographical structures considered as barriers were high mountains and large water bodies. Mountains higher than 1700 meters and water surfaces more than 200 kilometres from the nearest coast were considered as barriers. If adult birds migrate by a vector orientation program mortality from barrier crossing could potentially cause route convergence. We modelled the migration of such adults by assuming that barrier crossing would result in mortality and we terminated all simulated tracks from the point where a track reached a barrier. To be comparable with our tracked adults, we included tracks up until the point of termination, since we also have incomplete routes in the tracked birds.

#### Simulation including directional changes as response to barriers: model (iv)

Here, we assumed that a bird encountering a barrier would choose a new direction for a 100 km step (from the same von Mises distribution). After 100 unsuccessful attempts of finding a random direction that would lead it around the barrier, it was allowed to pass. This scenario imitates a situation where the birds follow a clock-and-compass system, but have an innate response to try to find a new direction when they face a barrier (except if going around the barrier would infer getting too far off course, in which case they cross it).

#### Simulation with removal of entire tracks reaching barriers: models (v) and (vi)

For models incorporating barriers, the assumptions differed between models of juvenile and adult migration. In scenarios (v) and (vi) we assume that the birds migrate by a vector orientation program only as juveniles, while survivors after the first journey (adults) stick to the same or a very similar route as they used as juveniles based on navigation cues that they have learned during the first journey. Assuming mortality of migrating juveniles when crossing barriers could cause convergence in adults. We can therefore directly compare the simulated routes avoiding barriers (i.e. we see only those not dying from barrier crossing) with the observed routes for adults. Under this scenario, we removed the complete tracks of those birds that cross a barrier both for the long and the short step lengths.

#### Evaluation of model fit to observed data

After all simulations, we calculated the distance between birds at each stopover site. We ran 10,000 bootstraps of n randomly drawn simulated routes and calculated the distance to the mean stopover position (n is the number of tracked birds we have positions for at the respective stopover). All simulations and bootstraps were done in R2.14.0 [36] using packages: “CircStats”, “geosphere”, “maptools” and “sp” [37], [38], [39], [40]. All route representations were done in ArcMap10 [41].

## Results

### Tracking

**Figure 1:**
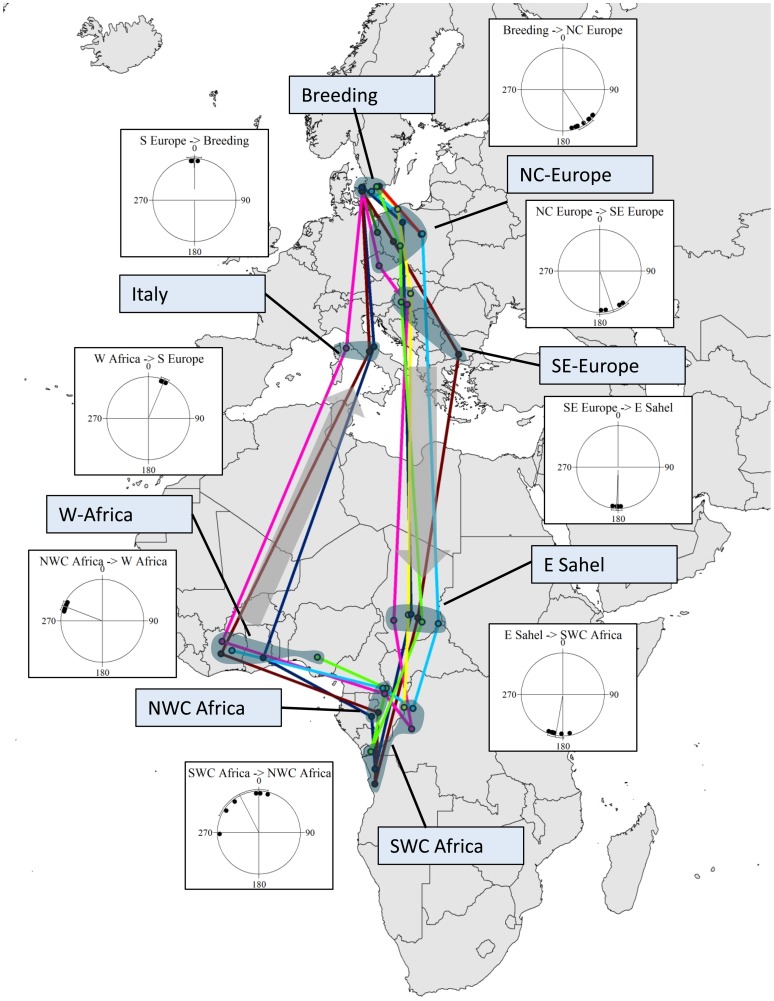
Staging areas of eight satellite-tracked adult common cuckoos with vector directions between stopovers indicated by inserted orientation diagrams. Lines are connecting staging sites and do not necessarily represent the paths followed (see ESM Fig. S1). Mercator projection.

The cuckoos followed very similar routes (Fig. 1) with average total distances travelled in autumn of 7118 km and in spring 9136 km (see Table 2 for a summary of migration timing, distance and speed). They stopped over for prolonged periods of time in north-central Europe (n = 8 out of 8 alive and transmitting individuals), south-eastern Europe (n = 4/7, in three birds it was not possible to determine if they used this stopover due to missing transmissions though), eastern Sahel (n = 6/6), south-western Central Africa in winter (n = 6/6), north-western Central Africa (n = 5/5), West Africa (n = 5/5) and southern Europe (n = 3/3). The migration corridor was remarkably narrow with average distances to the mean position on each stopover ranging from 123–456 km, all smaller than the 480 km in the over-wintering area. In the breeding area the average distance was 59 km before migration and 15 km after returning from migration (because only Danish birds returned). Birds crossed the Sahara in autumn in eastern Libya or western Egypt and Sudan (n = 4). In spring they moved north from West Africa into Mali and Niger and further through Algeria (n = 4), and presumably crossed the Mediterranean Sea between Tunisia and Italy (n = 3; see ESM Figure S1 and Table S1 for details on individual route, timing and stopover duration). The stopovers in north-central Europe lasted on average 26±20 days (mean±sd), in south-eastern Europe 26±19 days, in eastern Sahel 39±19 days, in the over-wintering site in south-western Central Africa 100±15 days, in north-western Central Africa 35±18 days, in West Africa 31±15 days, and in southern Europe 10±1 days before returning to the breeding sites after more than 10 months leaving only 49±3 days for the annual residence time in the breeding area. Two of the three individuals that were tracked for a complete migration cycle returned to the same breeding site, while the third bird moved to a site 8 km from where it was caught. The median return date (17/5) is rather late compared to literature data, but fits well with peak arrival this year (Fig S2).

**Table 2:** Summary table of the autumn and spring migration of eight adult common cuckoos tracked from southern Scandinavia.

Season	Bird	Migration period	Total distance (km)	Total time (days)	Average speed (km/d)	Total travelling days	Av. travelling speed (km/d)
Autumn	Male 19150†	12 Jul–2 Sep†	4158†	52†	80†	?	?
	Male 49466	8 Aug–14 Oct	6261	67	93	<16	>391
	Female 57372	4 Jul–18 Nov	7013	137	51	<27	>260
	Male 57374	1 Jul–2 Nov	6712	124	54	?	?
	Female 36328	18 Jul–22 Oct	6786	96	71	?	?
	Male 36331	6 Jul–19 Nov	8378	136	62	<36	>233
	Female 36332†	30 Jun–6 Sep†	398†	68†	6†	1†	398†
	Male 36487	30 Jun–5 Nov	7559	128	59	<23	>329
	Average‡	10 Jul–3 Nov	7118	115	65	26	303
Spring	Female 57372†	16 Feb–15 May†	5604†	88†	64†	<27†	>208†
	Male 57374†	5 Mar–1 May†	3752†	57†	66†	9†	417†
	Female 36328	30 Jan–2 Jun	9100	123	74	22	414
	Male 36331	26 Feb–17 May	9729	80	122	28	347
	Male 36487	28 Jan–9 May	8578	101	85	25	343
	Average‡	14 Feb–19 May	9136	101	94	25	368

†Transmitters stopped prior to migration completion.

‡Only complete migrations are included in the averages.

Distances are travelled distances along the routes followed, but not including movements within a staging area. Due to missing transmissions, the exact number of travel days was difficult to determine in several cases and the listed numbers are maxima (consequently travelling speeds are minima).

### Clock-and-compass simulations

**Figure 2:**
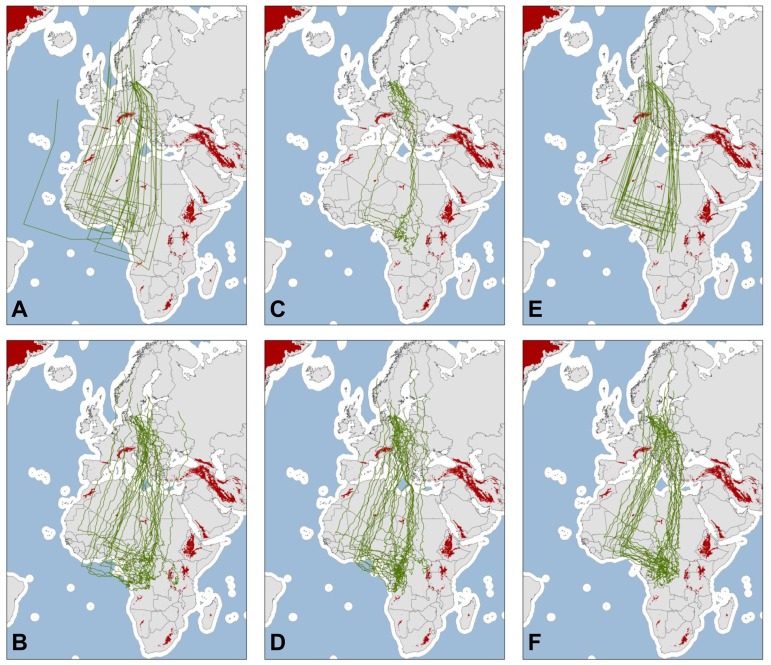
Maps showing examples of 15 random migration routes simulated by vector summation in six different ways. A: simulations using the LONG model, B: SHORT, C: SHORT_Mort, D: SHORT_Dir_Change, E: LONG_Juv_Mort, F: SHORT_Juv_Mort (see Table 1). Blue parts of maps indicate water bodies considered a barrier (>200 km from the coast) and red parts indicate mountain barriers (more than 1700 m above sea level). Mercator projection.

**Figure 3:**
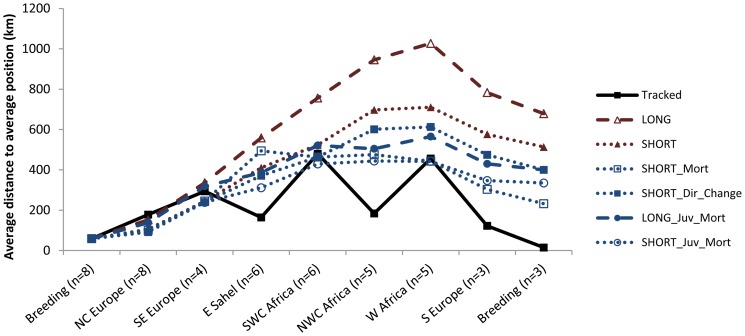
Distances among individual common cuckoos on tracked (solid line) and simulated stopovers during the whole annual migration (see **Table 1** for explanation of the types of simulations). In the tracked birds, the values represent average distance from the birds to the average position at that stopover. For the simulated birds, the values are averages of 10,000 bootstraps of average distance from the birds to the average position in that specific stopover. In NC Europe and SE Europe no simulations were significantly different from the tracked birds. In E Sahel, NWC Africa, S Europe and Breeding all simulations were significantly more scattered than the tracked birds. In SWC Africa and W Africa only LONG was significantly more scattered than the tracked individuals (statistical comparisons based on bootstraps in Table S2).

Tracks simulating a clock-and-compass migration system (Fig. 2) showed a significantly larger scatter than the observed tracks (Fig. 3; for statistical test values see Table S2). Independently of step length, the simulated routes diverged significantly more than the observed tracks after only three steps (Fig. 3 and ESM Table S2). When simulating smaller steps, the scatter of the simulated tracks was similar to the observed tracks in the winter site in southwest Central Africa (Fig. 3) but still larger at stopovers. Taking topography into account improved the model fit, though neither direct mortality nor avoidance of barriers caused the same degree of route convergence as observed in the tracked birds (Fig. 3). Similar patterns were found when assuming that the routes taken by juveniles using vector orientation with barrier-related mortality is reflected in the routes of adults (Fig. 3). Pure vector orientation is normally expected to be associated with a pattern of increasing scatter with increasing distance. However, this was not observed for the simulations after the birds' departure from West Africa in spring, which was due to the fact that (a) the simulations during the final part of spring migration were based on a reduced sample of only three very similar tracks and (b) the birds moved northwards and the absolute distances in frontal spread with a given angular spread in courses will be smaller for a movement from the equator and northwards compared to a corresponding southward movement towards the equator.

## Discussion

We found a very narrow migration corridor in a nocturnal, solitary migrant, and the tracks often converged on stopovers along the route. Since this contradicts the pattern expected from a pure clock-and-compass system, such a narrow corridor is highly surprising for a nocturnal solitary migrant relying solely on genetic information. Even though the overwintering range appeared relatively small, the distance between birds was largest in winter. Stopover habitat was primarily open farmland or wetlands (NC Europe, SE Europe, E Sahel, W Africa, and S Europe). The wintering sites and the north-western Central African spring stopover sites were situated in more forested habitats. We have tracked relatively few birds, and with more individuals tracked we would likely see some individuals with divergent routes, this will however most likely be few individuals and not alter the extent of the corridor to any large degree.

Observed routes did not fit the expectations from simple clock-and-compass migration scenarios, even if taking topography such as mountain and ocean barriers into account. Avoidance of geomorphological structures during vector summation reduced the spread in migration routes, but still observed tracks were more concentrated than simulated tracks, at least for some stopovers. Even allowing directional changes induced by barriers, as could be possible both for juvenile and adult birds, resulted in more scatter than was actually observed. That adults do not use vector orientation alone is according to theory, since they should be experienced and able to navigate [4], [9]. However, that a population of adult birds would have such a narrow migration corridor is unexpected and hard to explain by vector orientation among juvenile birds, as it implies that only the juveniles that followed the correct route during their first migration survived. Our simulation scenarios of juveniles migrating by vector summation followed by adult route replication were characterized by unreasonably high mortality costs (98%), as also found by Thorup and Rabøl [14] for cases of long-distance songbird migration (up to 90% in autumn migration alone), and still the scatter between locations of surviving individuals (simulations) were larger than that observed (Fig. 3).

A possible alternative explanation for the simulations to be insufficient in explaining route convergence is that the birds are influenced by additional factors such as winds, odour guidance or habitat leading lines, where specific habitat types could be attracting and possibly guiding birds (e.g. rivers or valleys). However, a more likely explanation is that the cuckoos could rely on true navigation. The fact that the tracks were extremely close to each other in for example southern Chad in autumn or Italy in spring suggests that the birds are navigating towards specific *en route* goal areas as proposed by Rabøl [13]. Finally, it cannot be completely excluded that some kind of social interactions could influence the routes chosen by the birds even though this appears unlikely given the mostly solitary behaviour observed in cuckoos during migration [23], and none of the birds tracked in this study were travelling together.

Whether vector summation is sufficient to explain observed migration patterns is a controversial question [33]. As the first two steps (within Europe) in this study's simulations were not significantly different from the observed, it points to the possible sufficiency of vector summation to explain observed patterns in some studies [12], [29] of intra-European migration (but see [33]). All three studies which concluded that vector summation is insufficient to explain the migration patterns observed [14], [42], [43] simulated long-distance migration into Africa, as in this study.

The route and stopover areas of the southern Scandinavian population of cuckoos are likely an adaptation to fit the spatio-temporally optimal route in relation to habitat, available resources, safe barrier crossing and dominating wind systems, the details of which are subject to future research. If so, even small changes in the breeding range could result in different routes being optimal and different populations even within Europe would have different migration routes and stopovers, also even if they share the winter range. The arrival to southern Chad for instance is very well timed with peak primary productivity following the rainy season. Comparable habitats with similar seasonal timing occur over most of the southern Sahel though, why other stopover areas might be more cost-effective for other populations. Indeed, most British cuckoos seem to migrate along different routes, indicating population specific migration patterns [44], although they follow a similar clockwise loop and winter in the same region. More detailed comparative studies of different populations are in progress. The existence of narrow migratory corridors and specific stopover areas point to the possible importance of conservation measures *en route* as e.g. stopover habitat degradation, high hunting pressures or collision risks will affect the entire population using a flyway [45], [46], [47], [48]. This is well-known within conservation of e.g. shorebirds, geese and raptors, but our results suggest that this should not be neglected in the smaller nocturnal migrants.

The documentation of a highly unexpected narrow migration corridor in a nocturnal migrant calls for further studies. A more experimental approach, involving displacements in space and time, is necessary to test whether young and adult cuckoos really navigate towards specific stopover sites (perhaps based on “sign-posts” of unknown nature) or whether they are guided by other cues related to the landscape.

## Supporting Information

Figure S1 **Tracks of eight individual common cuckoos as recorded by satellite telemetry.** For stationary birds, only the highest quality position from each 10 h transmission period was included. For travelling birds, all positions during the 10 h transmission periods were used for analyses (cf. Methods). Mercator projection.
(TIF)

Figure S2 **Timing of arrival of Common cuckoos in Denmark in 2011.** The phenology of new unique locations with observations of cuckoos in Denmark in 2011. Note the main peak of new locations (indicating massive arrival) is in mid May. Source: DOFbasen, www.dofbasen.dk, Dansk Ornitologisk Forening (BirdLife Denmark). Accessed 2013 Nov 27.
(TIF)

Table S1 **Timing of migration stages in eight adult common cuckoos tracked by satellite telemetry.** When departure or arrival was in a period of missing transmissions, the dates are not known exactly as indicated with “<” or “>”. The symbol “-” indicates that a bird stopped transmitting before that stopover, whereas “?” indicates that we have no positions for the bird at that stopover, but it could have used it during a period with missing transmissions. Step distance and step direction are the basis for the actual vectors in the first simulations, and are the distances and directions travelled from the preceding stopover to the current. Median/mean are medians of departure/arrival dates for the specific stopover, and means of durations, distances and directions for the specific stopover.
(DOCX)

Table S2 **Simulating distances between individuals.** Simulation results based on vector summation (10,000 bootstraps) of distances between individuals from the four different types of simulation compared with observed data for the cuckoos recorded by satellite tracking. Unshaded rows show the average distances between individuals ±standard deviation, and shaded rows show p-values for simulated routes to be lower than observed data.
(DOCX)
