# Supplementary figures and images for: Narrow-Front Loop Migration in a Population of the Common Cuckoo Cuculus canorus, as Revealed by Satellite Telemetry
Source: PLoS One. 2014 Jan 8;9(1):e83515. doi: 10.1371/journal.pone.0083515 (PMC3885432; doi:10.1371/journal.pone.0083515)

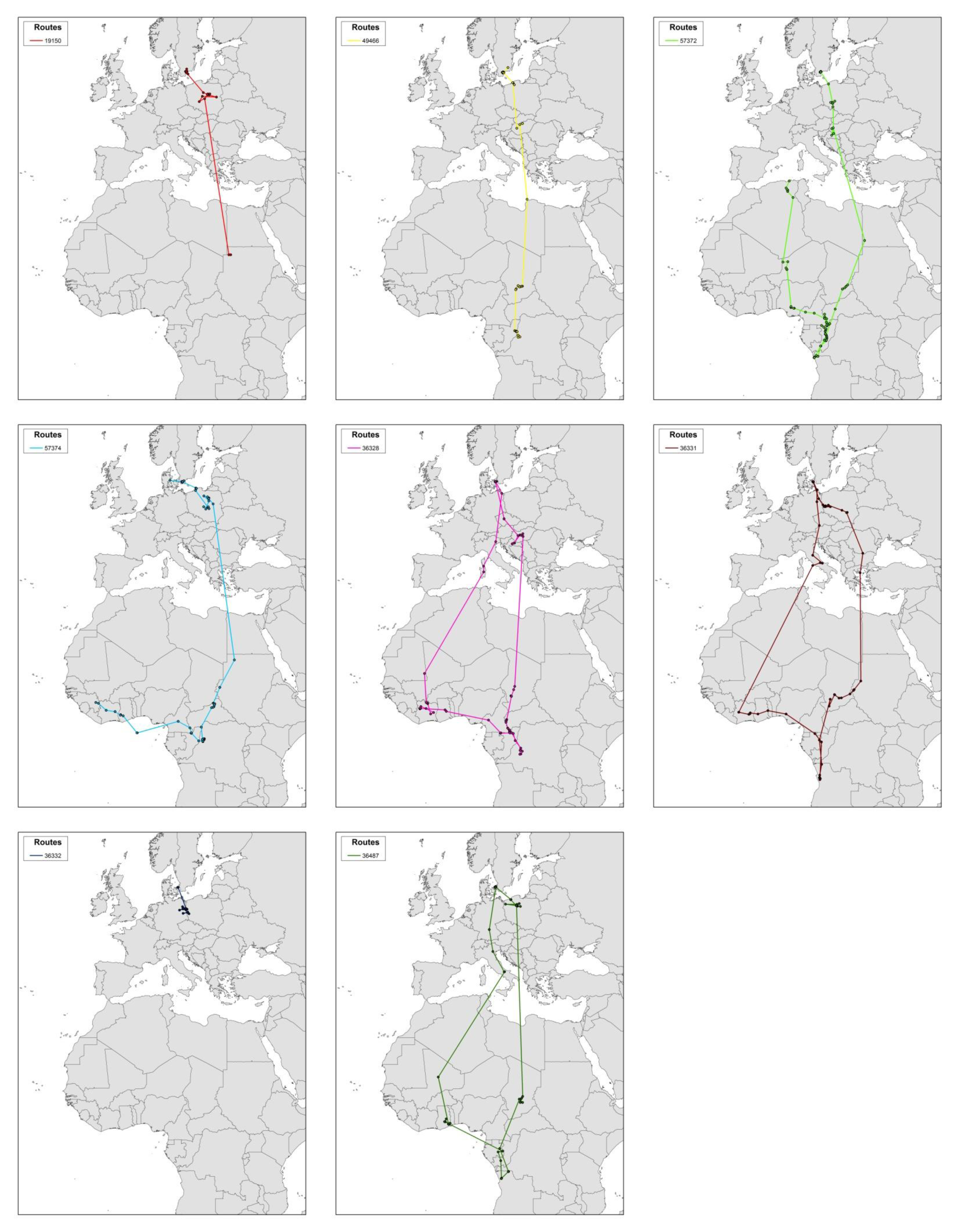

Supplement: Figure S1 — Tracks of eight individual common cuckoos as recorded by satellite telemetry. For stationary birds, only the highest quality position from each 10 h transmission period was included. For travelling birds, all positions during the 10 h transmission periods were used for analyses (cf. Methods). Mercator projection. (TIF) [file pone.0083515.s001.tif]

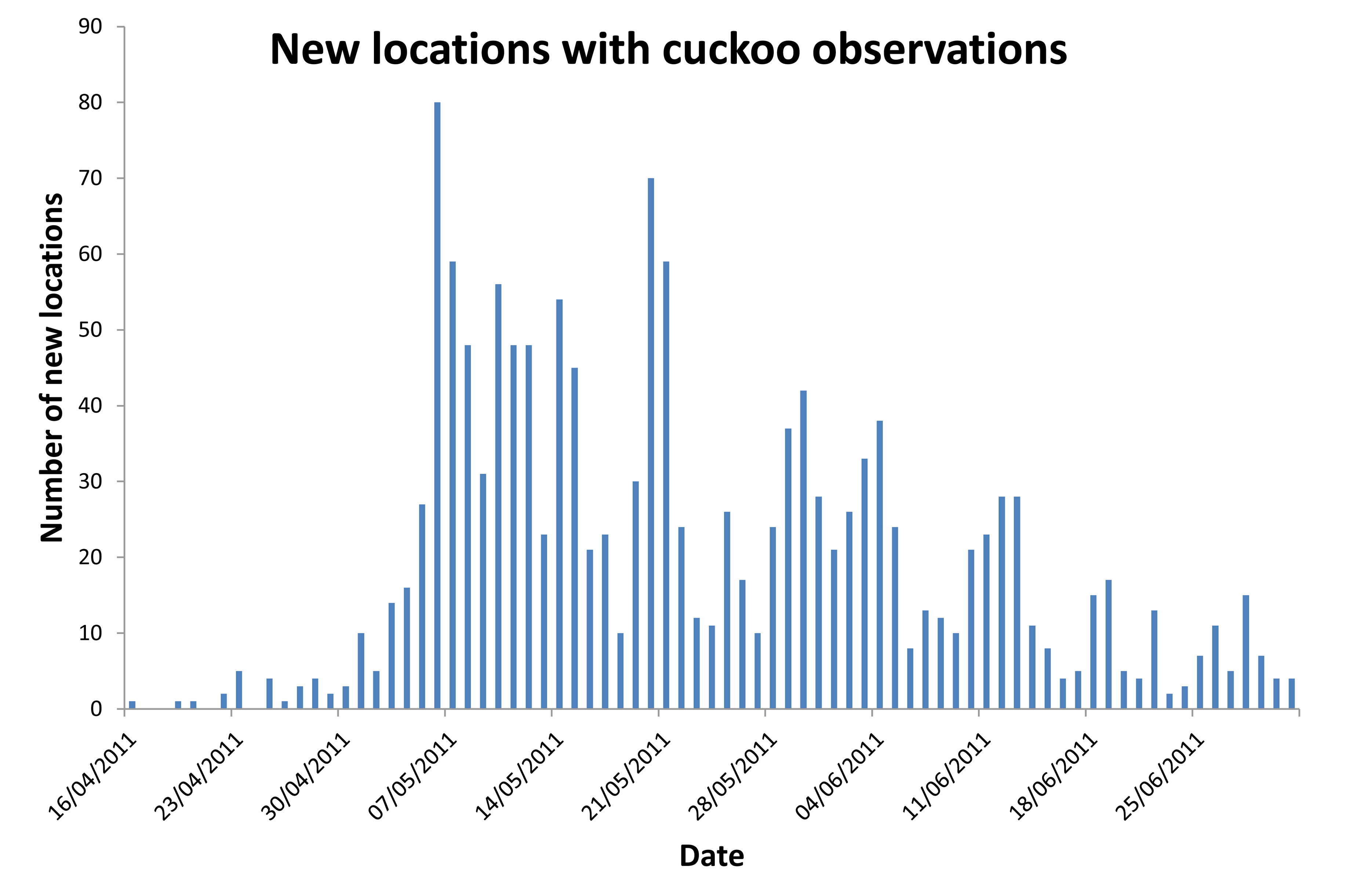

Supplement: Figure S2 — Timing of arrival of Common cuckoos in Denmark in 2011. The phenology of new unique locations with observations of cuckoos in Denmark in 2011. Note the main peak of new locations (indicating massive arrival) is in mid May. Source: DOFbasen, www.dofbasen.dk, Dansk Ornitologisk Forening (BirdLife Denmark). Accessed 2013 Nov 27. (TIF) [file pone.0083515.s002.tif]
