# Supplementary material for: Narrow-Front Loop Migration in a Population of the Common Cuckoo Cuculus canorus, as Revealed by Satellite Telemetry
Source: PLoS One. 2014 Jan 8;9(1):e83515. doi: 10.1371/journal.pone.0083515 (PMC3885432; doi:10.1371/journal.pone.0083515)
Supplement: Table S1 — Timing of migration stages in eight adult common cuckoos tracked by satellite telemetry. When departure or arrival was in a period of missing transmissions, the dates are not known exactly as indicated with “<” or “>”. The symbol “-” indicates that a bird stopped transmitting before that stopover, whereas “?” indicates that we have no positions for the bird at that stopover, but it could have used it during a period with missing transmissions. Step distance and step direction are the basis for the actual vectors in the first simulations, and are the distances and directions travelled from the preceding stopover to the current. Median/mean are medians of departure/arrival dates for the specific stopover, and means of durations, distances and directions for the specific stopover. (DOCX) [file pone.0083515.s003.docx]

| Tag Id | Stopover | Arrival | Departure | Duration | Step distance | Step direction |
| --- | --- | --- | --- | --- | --- | --- |
| Male 19150 | Breeding |  | 12-Jul |  |  |  |
| Male 49466 | Breeding |  | 08-Aug |  |  |  |
| Female 57372 | Breeding |  | 04-Jul |  |  |  |
| Male 57374 | Breeding |  | >30-Jun |  |  |  |
| Female 36328 | Breeding |  | 18-Jul |  |  |  |
| Male 36331 | Breeding |  | 06-Jul |  |  |  |
| Female 36332 | Breeding |  | 30-Jun |  |  |  |
| Male 36487 | Breeding |  | 30-Jun |  |  |  |
| Median/mean | Breeding |  | 06-Jul |  |  |  |
| Male 19150 | NC Europe | 13-Jul | >17-Jul | >5 | 536 | 139° |
| Male 49466 | NC Europe | 09-Aug | >18-Aug | >10 | 249 | 138° |
| Female 57372 | NC Europe | 06-Jul | 02-Aug | 28 | 542 | 158° |
| Male 57374 | NC Europe | <6-Jul | >27-Aug | >52 | 558 | 130° |
| Female 36328 | NC Europe | <25-Jul | >27-Jul | >3 | 664 | 167° |
| Male 36331 | NC Europe | 09-Jul | 12-Aug | 35 | 503 | 148° |
| Female 36332 | NC Europe | 01-Jul | - | - | 398 | 132° |
| Male 36487 | NC Europe | 01-Jul | >17-Aug | >48 | 442 | 162° |
| Median/mean | NC Europe | 07-Jul | 10-Aug | 26 | 487 | 147° |
| Male 19150 | SE Europe | ? | ? | ? | ? | ? |
| Male 49466 | SE Europe | <26-Aug | 15-Sep | 20 | 773 | 171° |
| Female 57372 | SE Europe | <8-Aug | >24-Sep | >47 | 531 | 179° |
| Male 57374 | SE Europe | ? | ? | ? | ? | ? |
| Female 36328 | SE Europe | <4-Aug | >6-Sep | >33 | 460 | 144° |
| Male 36331 | SE Europe | <26-Aug | 27-Aug | >2 | 1278 | 150° |
| Female 36332 | SE Europe | - | - | - | - | - |
| Male 36487 | SE Europe | ? | ? | ? | ? | ? |
| Median/mean | SE Europe | 17-Aug | 11-Sep | 26 | 760 | 161° |
| Male 19150 | E Sahel | - | - | - | - | - |
| Male 49466 | E Sahel | 19-Sep | 11-Oct | 23 | 3910 | 180° |
| Female 57372 | E Sahel | <9-Oct | 14-Nov | >37 | 3939 | 176° |
| Male 57374 | E Sahel | 28-Sep | >26-Oct | >29 | - | - |
| Female 36328 | E Sahel | 28-Sep | 18-Oct | 21 | 3888 | 182° |
| Male 36331 | E Sahel | 02-Sep | 06-Nov | 66 | 3371 | 189° |
| Female 36332 | E Sahel | - | - | - | - | - |
| Male 36487 | E Sahel | <3-Sep | 29-Oct | >57 | - | - |
| Median/mean | E Sahel | 23-Sep | 29-Oct | 39 | 3777 | 182° |
| Male 19150 | SWC Africa (winter) | - | - | - | - | - |
| Male 49466 | SWC Africa (winter) | 14-Oct | - | - | 1328 | 183° |
| Female 57372 | SWC Africa (winter) | 18-Nov | 16-Feb | 91 | 2001 | 201° |
| Male 57374 | SWC Africa (winter) | 02-Nov | 05-Mar | 125 | 1270 | 197° |
| Female 36328 | SWC Africa (winter) | 22-Oct | 30-Jan | 101 | 1579 | 171° |
| Male 36331 | SWC Africa (winter) | 19-Nov | 26-Feb | 100 | 2463 | 194° |
| Female 36332 | SWC Africa (winter) | - | - | - | - | - |
| Male 36487 | SWC Africa (winter) | 05-Nov | 28-Jan | 85 | 2282 | 193° |
| Median/mean | SWC Africa (winter) | 03-Nov | 16-Feb | 100 | 1821 | 190° |
| Male 19150 | NWC Africa | - | - | - | - | - |
| Male 49466 | NWC Africa | - | - | - | 1971 | 269° |
| Female 57372 | NWC Africa | 28-Feb | 21-Apr | 53 | 942 | 14° |
| Male 57374 | NWC Africa | 06-Mar | 01-Apr | 27 | 521 | 304° |
| Female 36328 | NWC Africa | 05-Feb | 01-Apr | 56 | 639 | 323° |
| Male 36331 | NWC Africa | 02-Mar | 15-Mar | 14 | 1031 | 3° |
| Female 36332 | NWC Africa | - | - | - | - | - |
| Male 36487 | NWC Africa | 02-Feb | 25-Feb | 24 | 760 | 356° |
| Median/mean | NWC Africa | 28-Feb | 01-Apr | 35 | 977 | 211° |
| Male 19150 | W Africa | - | - | - | - | - |
| Male 49466 | W Africa | - | - | - | - | - |
| Female 57372 | W Africa | <26-Apr | >4-May | >9 | 1081 | 294° |
| Male 57374 | W Africa | 04-Apr | - | - | 2223 | 284° |
| Female 36328 | W Africa | <8-Apr | 16-May | >39 | 2440 | 288° |
| Male 36331 | W Africa | 28-Mar | 26-Apr | 30 | 2407 | 291° |
| Female 36332 | W Africa | - | - | - | - | - |
| Male 36487 | W Africa | 07-Mar | 19-Apr | 44 | 1768 | 299° |
| Median/mean | W Africa | 28-Mar | 26-Apr | 31 | 1984 | 291° |
| Male 19150 | S Europe | - | - | - | - | - |
| Male 49466 | S Europe | - | - | - | - | - |
| Female 57372 | S Europe | - | - | - | - | - |
| Male 57374 | S Europe | - | - | - | - | - |
| Female 36328 | S Europe | 21-May | 29-May | 9 | 4052 | 23° |
| Male 36331 | S Europe | 01-May | 11-May | 11 | 4312 | 26° |
| Female 36332 | S Europe | - | - | - | - | - |
| Male 36487 | S Europe | 25-Apr | 05-May | 11 | 4227 | 20° |
| Median/mean | S Europe | 01-May | 11-May | 10 | 4197 | 23° |
| Male 19150 | Breeding | - |  |  | - | - |
| Male 49466 | Breeding | - |  |  | - | - |
| Female 57372 | Breeding | - |  |  | - | - |
| Male 57374 | Breeding | - |  |  | - | - |
| Female 36328 | Breeding | 02-Jun |  |  | 1478 | 6° |
| Male 36331 | Breeding | 17-May |  |  | 1506 | 357° |
| Female 36332 | Breeding | - |  |  | - | - |
| Male 36487 | Breeding | 09-May |  |  | 1489 | 356° |
| Median/mean | Breeding | 17-May |  |  | 1491 | 240° |
