# Supplementary material for: Narrow-Front Loop Migration in a Population of the Common Cuckoo Cuculus canorus, as Revealed by Satellite Telemetry
Source: PLoS One. 2014 Jan 8;9(1):e83515. doi: 10.1371/journal.pone.0083515 (PMC3885432; doi:10.1371/journal.pone.0083515)
Supplement: Table S2 — Simulating distances between individuals. Simulation results based on vector summation (10,000 bootstraps) of distances between individuals from the four different types of simulation compared with observed data for the cuckoos recorded by satellite tracking. Unshaded rows show the average distances between individuals ±standard deviation, and shaded rows show p-values for simulated routes to be lower than observed data. (DOCX) [file pone.0083515.s004.docx]

|  | Breeding | NC Europe | SE Europe | E Sahel | SWC Africa | NWC Africa | W Africa | S Europe | Breeding |
| --- | --- | --- | --- | --- | --- | --- | --- | --- | --- |
| Tracked | **59 (n=8)** | **178 (n=8)** | **294 (n=4)** | **164 (n=6)** | **480 (n=6)** | **185 (n=5)** | **456 (n=5)** | **123 (n=3)** | **15 (n=3)** |
| LONG | 59 | 152 ±30 | 336 ±110 | 558 ±142 | 756 ±160 | 946 ±263 | 1027 ±282 | 783 ±294 | 678 ±252 |
| LONG | 1.00 | 0.80 | 0.35 | 0.00 | 0.04 | 0.00 | 0.01 | 0.00 | 0.00 |
| SHORT | 59 | 105 ±25 | 243 ±75 | 411 ±111 | 524 ±131 | 698 ±184 | 710 ±187 | 577 ±216 | 513 ±193 |
| SHORT | 1.00 | 0.99 | 0.76 | 0.00 | 0.39 | 0.00 | 0.08 | 0.00 | 0.00 |
| LONG_Juv_Mort | 59 | 138 ±29 | 319 ±104 | 386 ±131 | 521 ±103 | 505 ±124 | 565 ±185 | 430 ±193 | 400 ±198 |
| LONG_Juv_Mort | 1.00 | 0.91 | 0.37 | 0.01 | 0.34 | 0.01 | 0.31 | 0.03 | 0.00 |
| SHORT_Juv_Mort | 59 | 95 ±65 | 239 ±139 | 311 ±201 | 429 ±252 | 444 ±279 | 442 ±291 | 347 ±223 | 335 ±202 |
| SHORT_Juv_Mort | 1.00 | 1.00 | 0.75 | 0.02 | 0.68 | 0.01 | 0.56 | 0.04 | 0.00 |
| SHORT_Mort | 59 | 100 ±23 | 245 ±78 | 494 ±106 | 464 ±114 | 475 ±115 | 443 ±122 | 303 ±118 | 232 ±107 |
| SHORT_Mort | 1.00 | 1.00 | 0.74 | 0.00 | 0.55 | 0.00 | 0.58 | 0.05 | 0.06 |
| SHORT_Dir_Change | 59 | 91 ±22 | 240 ±105 | 371 ±105 | 463 ±139 | 601 ±167 | 613 ±198 | 474 ±201 | 400 ±192 |
| SHORT_Dir_Change | 1.00 | 1.00 | 0.64 | 0.03 | 0.56 | 0.00 | 0.25 | 0.03 | 0.01 |
